# Supplementary material for: Effect of Solid Fat Content in Fat Droplets on Creamy Mouthfeel of Acid Milk Gels
Source: Foods. 2022 Sep 20;11(19):2932. doi: 10.3390/foods11192932 (PMC9563907; doi:10.3390/foods11192932)
Supplement: Supplementary file 1 [file foods-11-02932-s001.zip › foods-1881747-supplementary.pdf]

Table S1. Fatty acid composition of blended milk fats.

Fatty acid（%）	F10	F20	F40	F60	F85
Butyric acid(C4:0）	0.246±0.006^{c}	0.236±0.002^{c}	0.160±0.007^{b}	0.366±0.008^{d}	0.084±0.003^{a}
Caproic acid(C6:0）	0.302±0.021^{b}	0.271±0.000^{b}	0.469±0.027^{d}	0.413±0.021^{c}	0.096±0.003^{a}
Octanoic acid(C8:0）	0.215±0.005^{b}	0.227±0.001^{b}	0.395±0.025^{d}	0.359±0.003^{c}	0.089±0.001^{a}
Capric acid(C10:0）	0.576±0.038^{b}	0.649±0.002^{c}	1.112±0.046^{d}	1.045±0.002^{d}	0.194±0.013^{a}
Undecanoic acid(C11:0）	0.073±0.004^{a}	N.D.	N.D.	N.D.	N.D.
Lauric acid(C12:0）	0.903±0.004^{b}	1.034±0.002^{c}	1.728±0.011^{e}	1.687±0.000^{d}	0.367±0.023^{a}
Tridecanoic acid(C13:0）	0.031±0.001^{c}	0.024±0.001^{b}	0.047± 0.002^{d}	0.048±0.003^{d}	0.019±0.001^{a}
Tetradecanoic acid (C14:0)	3.267±0.038^{b}	3.693±0.001^{c}	6.000±0.015^{d}	6.127±0.003^{e}	1.968±0.029^{a}
Myristoleic acid(C14:1n5)	0.257±0.007^{b}	0.278±0.001^{c}	0.471±0.011^{d}	0.465±0.008^{d}	0.084±0.002^{a}
Pentadecanoic acid (C15:0)	0.335±0.001^{b}	0.364±0.003^{c}	0.601± 0.002^{d}	0.609±0.013^{d}	0.167±0.004^{a}
Cetylic acid (C16:0)	17.888±0.032^{a}	27.994±0.021^{b}	36.639±0.090^{c}	43.886±0.032^{d}	54.146±0.317^{e}
Zoomaric acid (C16:1n7)	0.478±0.012^{b}	0.476±0.003^{b}	0.716±0.002^{c}	0.706±0.002^{c}	0.123±0.004^{a}
Heptadecanoic acid (C17:0)	0.282±0.008^{b}	0.304±0.005^{c}	0.344±0.002^{d}	0.368±0.001^{e}	0.194±0.004^{a}
Stearic acid (C18:0)	6.389±0.097^{a}	14.166±0.009^{b}	18.628±0.023^{c}	24.511±0.033^{d}	39.699±0.352^{e}
Elaidic acid(C18:1n9t)	0.657±0.015^{c}	0.609±0.019^{b}	0.895±0.006^{d}	0.863±0.013^{d}	0.210±0.015^{a}
Oleic acid(C18:1n9c)	22.243±0.134^{e}	16.998±0.009^{d}	14.378±0.006^{c}	10.825±0.009^{b}	1.663±0.022^{a}
Linoleic acid(C18:2n6c)	39.760±0.109^{e}	28.116±0.008^{d}	14.575±0.016^{c}	6.071±0.012^{b}	0.144±0.006^{a}
α-Linolenic acid(C18:3n3)	4.805±0.052^{e}	3.473±0.010^{d}	1.908±0.006^{c}	0.874±0.007^{b}	0.051±0.003^{a}
Arachidic acid (C20:0)	0.306±0.001^{a}	0.332±0.005^{bc}	0.315±0.006^{ab}	0.336±0.008^{c}	0.493±0.011^{d}
Eicosaenoic acid(C20:1)	0.302±0.008^{d}	0.214±0.015^{c}	0.119±0.001^{b}	0.060±0.003^{a}	N.D.
Eicosadienoic acid(C20:2)	0.052±0.000^{b}	0.037±0.002^{b}	0.029±0.001^{a}	0.031±0.002^{a}	N.D.
Eicosatrienoic acid (C20:3n6)	0.026±0.001^{b}	0.023±0.000^{a}	0.033±0.00^{c}	0.035±0.001^{c}	N.D.
Eicosapentaenoic acid(C20:5n3)	N.D.	N.D.	0.044±0.001^{b}	0.035±0.000^{a}	N.D.
Arachidonic acid(C20:4n6)	0.029±0.000^{a}	0.040±0.001^{b}	0.041±0.000^{b}	0.039±0.002^{b}	N.D.
Heneicosanoic acid (C21:0)	0.059±0.000^{c}	0.039±0.001^{b}	0.040±0.002^{b}	0.029±0.000^{a}	N.D.
Docosanoic acid (C22:0)	0.328±0.002^{d}	0.240± 0.010^{c}	0.182±0.005^{b}	0.108±0.005^{a}	0.094±0.000^{e}
Tricosanoic acid (C23:0)	0.066±0.002^{d}	0.049±0.001^{c}	0.037±0.003^{b}	0.028±0.001^{a}	0.030±0.002^{a}

Tetracosanoic acid (C24:0)	0.126±0.006[e]	0.113±0.001[d]	0.096±0.000[c]	0.076±0.002[a]	0.086±0.002[b]
Saturated fatty acid	31.392±0.265[a]	49.736±0.065[b]	66.720±0.267[c]	79.995±0.135[d]	97.725±0.766[e]
Unsaturated fatty acids	68.608±0.339[e]	50.264±0.067[d]	33.208±0.052[c]	20.004±0.059[b]	2.275±0.050[a]

Results are mean ± SD (n = 3).

For each line, different letters indicate significantly different values ($P < 0.05$).

N.D. means not detected in sample.
